# Supplementary material for: Patterns of Intron Gain and Loss in Fungi
Source: PLoS Biol. 2004 Nov 30;2(12):e422. doi: 10.1371/journal.pbio.0020422 (PMC532390; doi:10.1371/journal.pbio.0020422)
Supplement: Table S1 — Also available at http://genes.mit.edu/NielsenEtAl/. (4.3 MB ZIP). [file pbio.0020422.st001.zip › NielsenEtAl/html/50.html]

AN4993.1.NCU02417.1.MG03561.1.FG06376.1


```
 CLUSTAL W (1.82) Multiple Sequence Alignments - Introns Inserted


Sequence 1: NCU02417.1	213 aa
Sequence 2: MG03561.1	250 aa
Sequence 3: FG06376.1	186 aa
Sequence 4: AN4993.1	213 aa
Alignment Length: 273 aa
Number Identitical Residues: 98 aa
Alignment Score (without introns) 4451


MG03561.1 	--------~--MAAVATVTSSNPPIDAGRVEKLLGELKYLQYEHGLEKEYLPSIRALISK
NCU02417.1	MAYWIDCT0LPIDMETTTEKAIVGTEVPHVEELPQELQYIKYEHKLEAEYLPAIRALISK
FG06376.1 	--------~----MATTKT-----------ENLPGELRYVQYEHALEAQYLPAIRALISK
AN4993.1  	--------~----MASTTHRP---------FDDPSSIRYIRYNPAHEDAYVAAMRQLISK
          	               :*   .          .   .::*::*:   *  *:.::* ****

MG03561.1 	DLSEPYSIYVYRYFLYQWGHLCFM0ALNPSDDS----LVGVIICKLEVHQSHSPPTRRGY
NCU02417.1	DLSEPYSIYVYRYFLYQWGHLCFM0ALHPVDSS----LIGVIICKLEPHASHSPPTLRGY
FG06376.1 	DLSEPYSIYVYRYFLCQWAHLCFM0ALNPVDSS----LIGVIVCKLEIHASHSNPTRRGY
AN4993.1  	DLSEPYSIYVYRYFLYQWGDLCFL~AMDTNSTTGEEQMIGVVVSKLEPHR-HGP--MRGY
          	*************** **..***: *:.. . :....::**::.*** *  *.    ***

MG03561.1 	IAMLAVSESFRGHGIATALVKQAIEAMAARDADE~IVLETEETNLSAMRLYERLGFMRSK
NCU02417.1	IAMLAVSSQHRGHGIATELVRRAIDAMAQRDADE~IVLETEETNIPAMRLYERLGFVRSK
FG06376.1 	IAMLAVASDFRGHGIATTLVKKAIDAMTKRNADE~IALETEETNVAAMRLYEQLGFLRTK
AN4993.1  	IAMLAVQEEYRGKGVATKLVRMAIDAMEKRGADE0IALETEITNTAAMKLYERLGFLRSK
          	****** ...**:*:** **: **:**  *.*** *.**** ** .**:***:***:*:*

MG03561.1 	KLHRYYLNGNSAYRLVLLLKH--------IDPDAAT-DLALDENDTR2LRGLLVYYNEMD
NCU02417.1	KLHRYYLNGNSAYRLVLLLRS--------VDADATN-DYAPDDRDIA~LR----------
FG06376.1 	KLHRYYLNGNSAYRLVLPLKY--------IDLDAG-----PNDLEII~NV----------
AN4993.1  	RLHRYYLNGNSAYRLVLYLKEGVGLMRTSFDPYAPPPDYAHDHLPPP~PALPAPLLSENG
          	:**************** *: . .   ::.*  *  .. : :.             .. .

MG03561.1 	ASFHFIIKSRRSRDTA1GCIEQIVIRAYVNGDWDKIRI
NCU02417.1	----------------~---------------------
FG06376.1 	----------------~---------------------
AN4993.1  	A---------------~---------------------
          	:
```
